# HPV vaccination and cervical cancer prevention in Pakistan: Why are we so far behind?

**DOI:** 10.1097/MS9.0000000000004964

**Published:** 2026-04-20

**Authors:** Areeba Zaidi, Fatima Kameleen, Muddassir Syed Saleem, Ahmed Asad Raza, Abedin Samadi

**Affiliations:** a Department of Medicine, Karachi Medical and Dental College, Karachi, Pakistan; b Department of Medicine, Jinnah Sindh Medical University, Karachi, Pakistan; c Department of Medicine, Kabul University of Medical Sciences Abu Ali Sina, Kabul, Afghanistan

**Keywords:** cervical cancer, HPV vaccination, human papillomavirus, Pakistan, public health policy

## Abstract

Cervical cancer remains a major global health challenge, disproportionately affecting women in low- and middle-income countries, largely due to persistent infection with high-risk human papilloma virus (HPV) types 16 and 18. In Pakistan, cervical cancer contributes substantially to female cancer morbidity and mortality, yet HPV vaccination coverage, awareness, and uptake remain critically low. Despite the proven effectiveness of HPV vaccines and screening in preventing cervical cancer, Pakistan faces significant social, logistical, and health system barriers that limit impact. Strengthening integration of HPV vaccination into national immunization programs, expanding culturally sensitive awareness initiatives, improving screening services, and adopting inclusive strategies – such as male vaccination – are essential to meet WHO cervical cancer elimination targets.

Cervical cancer (CC) ranks as the fourth leading cause of cancer-related death among women worldwide, chiefly driven by high-risk human papilloma virus (HPV) types 16 and 18. Its incidence and fatality are significantly higher in low- and middle-income countries than in developed regions^[^1^]^. Advanced CC may result in serious, potentially fatal complications such as severe bleeding, obstructive renal failure, pelvic organ fistulas, metastasis to distant organs, chronic pain, anemia, and sepsis^[^1^]^.

## Current burden of Cervical Cancer in Pakistan

In Pakistan, CC accounts for 5008 incident cases and 3197 fatalities annually, with high-risk HPV types 16 and 18 underpinning 88% of invasive malignancies^[^2^]^. In Asia, annual reports approximate 500000 incident cervical cancer cases with 250 000 resultant fatalities. Within Asia, cervical cancer incidence varies widely across countries. For example, Iraq reports one of the lowest estimated incidences (1.4 per 100 000 women), although this variation may reflect differences in screening coverage, health system reporting, and population-level risk factors rather than vaccination programs alone^[^3^]^. CC is substantially preventable through targeted immunization and robust screening interventions. Three HPV vaccines – bivalent, quadrivalent, and 9-valent – provide protection against the most oncogenic HPV strains. The most effective vaccination age is between 9 and 14 years, with studies showing that a 3-dose regimen for individuals aged 9–26 offers optimal protection. Three doses of the vaccine can nearly eliminate the risk of CIN-3, while recent findings suggest that a single dose may provide long-lasting protection for up to 8 years^[^3^]^.

## Barriers to HPV vaccination and prevention

Pakistan’s efforts to prevent cervical cancer and vaccinate against HPV face numerous logistical, social, and systemic obstacles. In 2024, Pakistan initiated a national HPV vaccination campaign targeting girls aged 9–14 years, representing an important step toward integrating HPV prevention within the national immunization framework^[^4^]^, but HPV vaccination in Pakistan is still extremely low, with 3.1% coverage, 20% awareness, and <10% uptake^[^5^]^. Access is largely limited to families able to afford private healthcare, thereby reinforcing inequities in vaccine availability. Vaccine hesitancy remains a major barrier to HPV vaccine uptake in Pakistan. Studies have identified multiple drivers including limited awareness of HPV, concerns about vaccine safety, misconceptions regarding infertility, and sociocultural sensitivities surrounding sexually transmitted infections^[^6,7^]^. Distrust in public health initiatives following misinformation during previous vaccination campaigns, particularly polio and measles eradication programs, has further amplified skepticism toward newer vaccines^[^8,9^]^. These factors collectively contribute to low acceptance of HPV vaccination among parents and adolescents. Persistent myths about infertility and distrust in public health programs turn a preventable disease into a silent threat, leaving countless women at risk of cervical cancer. Furthermore, inadequate integration between HPV vaccination services, cervical cancer screening, and follow-up care has produced a fragmented and ineffective prevention framework. Collectively, persistent deficiencies in awareness, access, and health system coordination continue to prevent HPV vaccination from reaching the majority of eligible girls in Pakistan.

## Policy recommendations for strengthening prevention

CC screening coverage in Pakistan remains extremely limited, with estimates suggesting that fewer than 5% of eligible women have ever undergone screening through Pap smear, visual inspection with acetic acid (VIA), or HPV testing, highlighting a major gap in preventive care. To strengthen HPV vaccination and cervical cancer prevention in Pakistan, urgent multi-sectoral policy action is required. Federal and provincial health authorities should fully integrate the HPV vaccine into the Expanded Program on Immunization, with long-term financing and logistics planning to ensure delivery beyond the initial campaign phase and equitable access across urban, rural, and underserved regions. To combat disinformation, eliminate stigma, and enhance understanding of vaccination safety and the relationship between HPV and cervical cancer, national and local governments must expand culturally responsive awareness programs in collaboration with schools, community leaders, and trusted health workers. Encouraging healthcare professionals to recommend the vaccine will increase uptake and trust proactively. Routine cervical cancer-screening facilities, such as Pap smears, VIA, and HPV testing, should be strengthened alongside vaccination efforts, with clear referral paths for precancerous lesions. Engagement with civil society and internet outreach can help to mitigate hesitation at the grassroots level. Furthermore, immunizing males curtails HPV transmission, thereby indirectly protecting females. Achieving the WHO’s 2030 target of 90% coverage by age 15 necessitates an urgent, coordinated, evidence-based public health approach to align Pakistan with global cervical cancer elimination targets^[^10^]^.

## AI transparency statement

To ensure methodological transparency and ethical compliance in manuscript preparation, we adhered to the recently developed TITAN (Transparency in the Reporting of Artificial Intelligence) 2025 guidelines, which outline standards for responsible AI use in biomedical writing and publishing^[^11^]^.

## Data Availability

No datasets were generated or analyzed during the current study.

## Gaurantor

Muddassir Syed Saleem.
